# DNA Methylation Profile of *β-1,3-Glucanase* and *Chitinase* Genes in Flax Shows Specificity Towards *Fusarium Oxysporum* Strains Differing in Pathogenicity

**DOI:** 10.3390/microorganisms7120589

**Published:** 2019-11-20

**Authors:** Wioleta Wojtasik, Aleksandra Boba, Marta Preisner, Kamil Kostyn, Jan Szopa, Anna Kulma

**Affiliations:** 1 Department of Genetic Biochemistry, Faculty of Biotechnology, University of Wroclaw, Przybyszewskiego 63, 51-148 Wroclaw, Poland; aleksandra.boba@uwr.edu.pl (A.B.); marta.preisner@upwr.edu.pl (M.P.); kamil.kostyn@upwr.edu.pl (K.K.); szopa@ibmb.uni.wroc.pl (J.S.); anna.kulma@uwr.edu.pl (A.K.); 2 Department of Genetics, Plant Breeding and Seed Production, Faculty of Life Sciences and Technology, Wroclaw University of Environmental and Plant Sciences, pl. Grunwaldzki 24A, 50-363 Wroclaw, Poland

**Keywords:** flax, *Fusarium oxysporum*, pathogenic and non-pathogenic strains, sensitization, DNA methylation, PR genes

## Abstract

Most losses in flax (*Linum usitatissimum* L.) crops are caused by fungal infections. The new epigenetic approach to improve plant resistance requires broadening the knowledge about the influence of pathogenic and non-pathogenic *Fusarium oxysporum* strains on changes in the profile of DNA methylation. Two contrasting effects on the levels of methylation in flax have been detected for both types of *Fusarium* strain infection: Genome-wide hypermethylation and hypomethylation of resistance-related genes (*β-1,3-glucanase* and *chitinase*). Despite the differences in methylation profile, the expression of these genes increased. Plants pretreated with the non-pathogenic strain memorize the hypomethylation pattern and then react more efficiently upon pathogen infection. The peak of demethylation correlates with the alteration in gene expression induced by the non-pathogenic strain. In the case of pathogen infection, the expression peak lags behind the gene demethylation. Dynamic changes in tetramer methylation induced by both pathogenic and non-pathogenic *Fusarium* strains are dependent on the ratio between the level of methyltransferase and demethylase gene expression. Infection with both *Fusarium* strains suppressed methyltransferase expression and increased the demethylase (*demeter*) transcript level. The obtained results provide important new information about changes in methylation profile and thus expression regulation of pathogenesis-related genes in the flax plant response to stressors.

## 1. Introduction

Currently, the greatest losses in flax crops are caused by fungal infections. Fungal diseases are the cause of about 20% of losses of flax cultivation. They result in the reduction of crop yield, both the seed and fiber and deterioration of their quality, as well as feed and food obtained from them [1]. Among the genus *Fusarium*, saprophytic *Fusarium oxysporum* is the major flax pathogen. The strains of *Fusarium oxysporum* can be divided depending on their virulence into pathogenic and non-pathogenic strains. The first group infects various plant species causing wilt or root rot and finally leads to death of the plant. By contrast, the latter do not invade the vascular system of plants and do not kill the host. Moreover, non-pathogenic strains of *F. oxysporum* colonizing plants without symptoms of fusariosis can protect the host from infection by pathogenic strains [2,3]. Therefore, some selected, non-pathogenic strains are perceived as potential biological control agents because their resistance-inducing activity correlates with an increase in the activity of pathogenesis-related (PR) proteins: β-1,3-glucanase and chitinase [4,5].

PR proteins play a very important role in the plant response to pathogenic infections. They are locally accumulated in infection sites and surrounding tissues, as well as in uninfected tissues, providing resistance to plants against subsequent infection [6]. β-1,3-glucanase is responsible for the degradation of the fungal cell wall by the hydrolysis of β-1,3-glycosyl bonds in β-1,3-glucan, thereby releasing the fungal cell wall fragments (oligosaccharides), which act as elicitors (damage-associated molecular patterns, DAMPs) in a defensive reaction stimulating the production of other PR proteins and antifungal low molecular weight components (phytoalexins) [7]. Chitinases catalyze the cleavage of bonds between C1 and C4, two residues of N-acetyl-D-glucosamine in chitin, inhibiting the growth of fungal hyphae during the invasion of the intercellular space, and releasing fungal elicitors (chitooligosaccharides or chitin oligomers) that induce a plant’s defense response by biosynthesis of Chitinases and other PR proteins [8].

There is a significant increase in the expression of both *β-1,3-glucanase* and *chitinase* genes during the infection, as confirmed in many plants, e.g., tomato, tobacco, soybeans, wheat, and peas [9,10]. At present, genetic engineering tools are frequently and routinely used to improve the plant defense system and thus plants’ resistance against pathogens. The results of such an approach are genetically modified organisms (GMOs). One possibility to strengthen the plant defense system making it pathogen-resistant is upregulation or overexpression of pathogen-related genes or genes encoding enzymes involved in the synthesis of secondary metabolites [11,12,13].

A similar effect, intense synthesis of pathogen-related proteins, was observed in various plant species after plant treatment with a non-pathogenic strain of *F. oxysporum* when compared to the control plants [3,14]. This was followed by an increase in the resistance against pathogenic strains of *F. oxysporum* in cucumber and peas [3]. In contrast, a study conducted by Aime and co-workers revealed that colonization of tomato by a non-pathogenic strain resulted in a decreased mRNA level of *β-1,3-glucanases* and *chitinases* genes. Furthermore, the expression of this group of genes was observed only when tomato plants subsequently underwent infection with a pathogenic strain of *F. oxysporum*. Such response, called priming, implies the protective role of non-pathogenic strains by accelerating and strengthening the plant defense mechanisms only upon contact with the pathogen [15].

After exposure to non-pathogenic fungal strains, the plants develop acquired immunity to pathogenic infections. Moreover, in this way the immune memory associated with the process of preparing cells (priming) may be transferred to the next generation. Priming results from the accumulation in the cell of inactive mitogen-activated protein kinases (MPK3, MPK6), and nonexpressor of pathogenesis-related genes 1 (NPR1) (both mRNA and protein) that are activated after infection [16]. Priming also affects chromatin modifications: Mostly methylation and acetylation of histones and DNA, associated with activation of genes involved in plant resistance. A local pathogenic infection can change the status of methylation and acetylation of histones and in particular can affect the promoter sequences of genes in systemic tissues. Such epigenetic changes provide plants with a long-term immune memory inherited from generation to generation [17].

Among eukaryotes, the plant genome bears the highest levels of DNA methylation, with up to 50% in some species. DNA methylation and modification of associated chromatin proteins determines the epigenetic status of the genome. Depending on the sequence context of the cytosine residue to be methylated, we can define CG, CNG, or CNN (C = cytosine; G = guanine; N = nucleotide other than G) specific methylation types. Two types of maintenance methyltransferase exist in plants: DNA methyltransferase (MET1), which predominantly methylates CG sites, and plant-specific chromomethylase 3 (CMT3) that methylates CNG (N = A, C, or T) sites. The third type methylates CNN and is controlled by domains of rearranged methyltransferase 1 (DRM2), a homologue of mammalian de novo DNA methyltransferase DNMT3 [18]. De novo DNA methylation is conducted by the pathway that includes specific transcripts that are copied into dsRNA by RNA-dependent RNA polymerase 2 (RDR2). This siRNA associates with argonaute 4 (AGO4) in a complex that mediates DNA methylation [19].

Our preliminary data indicate that single base pair mapping (bisulfite-seq) of methylated cytosine in flax seedlings reveals that over 8% of CG, 4% of CNG, and 7.3% of CNN is methylated. For comparison, the methylation state of maize genome (mainly composed of repetitive elements) ranges around 75% and 62% of CG and CNG sequence contexts, respectively [20]. Genome-wide mapping of methylated cytosine in *Arabidopsis thaliana* (*A. thaliana*) revealed levels of 24% CG, 6.7% CNG, and 1.7% CNN methylation [21].

Many literature reports have described changes in DNA at the genome-wide level caused by environmental stress, which can be inherited [22]. Pathogenic infection and abiotic stress can lead to two contrasting effects at the level of methylation in plants: Hypermethylation at the genome-wide level and hypomethylation of resistance-related genes, which can correlate with regulation of the expression of the defense-related genes in plants [23].

Based on the current state of knowledge about the mechanisms of epigenetic inheritance, it is anticipated that changes in the flax epigenome introduced by infection with a non-pathogenic *Fusarium oxysporum* strain would be directional and transmitted to the offspring. The aim of the study is to accurately understand and compare the mechanisms of infection of pathogenic and non-pathogenic strains as well as the sensitizing effect of the non-pathogenic strain, and in particular to examine the DNA methylation not only throughout the genome but also in specific genes. PR proteins are essential to plant resistance to biotic stress, which makes them the main focus of this research. The findings of our research can help to improve crop resistance in the future.

## 2. Materials and Methods

### 2.1. Plant and Fungal Material

A fibrous flax variety (*Linum usitatissimum* L. cv. Nike) was cultured under strictly defined conditions: Medium: MS (Murashige and Skoog, 1962) (Sigma-Aldrich, Saint Louis, Missouri, USA) with 0.8% agar (Sigma-Aldrich, Saint Louis, Missouri, USA) and 1% sucrose (Chempur, Piekary Slaskie, Poland); humidity: 50%; temperature: 21 °C/16 °C; illuminance: 23 mmol/s/m^3^; day/night: 16/8 h. The pathogenic strain *Fusarium oxysporum* f. sp. *linii* (Bolley) Snyder et Hansen (ATCC number: MYA-1201, Foln3) and the non-pathogenic strain *Fusarium oxysporum* (ATCC number: MYA-1198, Fo47) were bought from ATCC (Manassas, Virginia, USA) cultured on PDA (potato dextrose agar) medium (Sigma-Aldrich, Saint Louis, Missouri, USA) at 28 °C in darkness.

Molecular analysis (Figure S1): Seed germination, and growth of seedlings were carried out on Petri dishes under controlled conditions in a phytotron. Seven days after sowing, the seedlings were transferred (together with medium) to the PDA medium with either a pathogenic or non-pathogenic strain of *Fusarium oxysporum*, or to a PDA medium (control). Fungal strains were earlier grown on PDA medium for 3 and 5 days, respectively. Seedlings were harvested at the 6^th^, 12^th^, 24^th^, 36^th^, and 48^th^ hour of incubation (100 for each stage), frozen in liquid nitrogen, stored at -70 °C, and powdered before use. Treated flax seedlings were prepared in three biological repetitions. Each treatment had its own control. In order to sensitize the flax plants with the strain Fo47, first flax seedlings were incubated with Fo47 for 12 h. Twelve hours was chosen as the optimal sensitization time because after this period we observed the largest increase in the expression levels of *β-1,3-glucanase 1* and *chitinase* in preliminary studies. Subsequently, plants were transferred and incubated with pathogenic Foln3. For controls, plants were grown for 12 h on PDA medium without fungi then transferred to new PDA medium also without fungi. After 6, 12, 24, 36, and 48 h, seedlings were collected separately.

Phenotypic analysis: Four-week-old flax plants (from in vitro culture) were transferred onto a PDA medium with non-pathogenic strains of *F. oxysporum*, pathogenic strains of *F. oxysporum,* and without fungi (control plants) for two days. Before use, *F. oxysporum* strain was grown on PDA medium for 5 days. Next, flax plants incubated with non-pathogenic strain of *F. oxysporum* were transferred onto pathogenic *F. oxysporum* or non-pathogenic strain of *F. oxysporum*, and control plants were transferred to pathogenic strain of *F. oxysporum* or a PDA medium without fungi. Flax plants incubated with pathogenic strain of *F. oxysporum* for two days were transferred onto pathogenic *F. oxysporum.* Phenotypic changes were observed after four and six days.

### 2.2. Identification of DNA Sequences

In order to find the complete coding sequences of the genes, we searched the flax genome (Acc. No. AFSQ00000000.1) using fragments of DNA/cDNA obtained previously in our laboratory, via homology search and PCR, to obtain clones containing the genomic sequence of the GOI (gene of interest). Flax clones were scanned using FGENESH, SoftBerry (Mount Kisco, NY, USA). The resulting coding sequence was compared to the sequences from different plant species available in NCBI using BLAST software.

### 2.3. DNA Isolation

Genomic DNA was isolated using DNeasy Plant Mini Kit (QIAGEN, Hilden, Germany) following the manufacturer’s protocol. The DNA integrity was examined by gel electrophoresis on 1.0% (w/v) agarose and the DNA amount was checked by spectrophotometry method (Spectrophotometer Implen NanoPhotometer Pearl, München, Germany) at 260 nm.

### 2.4. Determination of Methylation Patterns of Genes

The methylation pattern of DNA was determined using the combination of restricted cleavage of the gene sequence at methylation sites (CCGG islands) using the restriction enzymes *MspI* and *HpaII* (New England Biolabs, Ipswich, Massachusetts, USA) and real-time PCR using primers flanking the methylation islands (cleavage sites). The difference between these two isoschizomers is that *HpaII* can cleave unmethylated CCGG, but *MspI* can cleave unmethylated CCGG as well as the sequence when the internal C residue is methylated CCmGG. Both of these enzymes do not cleave the sequence CCGG when the external and internal C is methylated (CmCmGG).

The reaction (in final volume 50 µL) of restriction cleavage by *HpaII* (250 units) and *MspI* (250 units) was run at 37 °C overnight on the template DNA (0.75µg), which later served as a template for real-time PCR reactions. Restrictions enzymes were inactivated by incubation at 80 °C for 20 min. The program used for real-time PCR was 95 °C for 10 min, and 40 cycles of denaturation for 10 s at 95 °C, annealing for 20 s at 57 °C, and extension for 25 s at 72 °C, and reaction mixtures contain (in final volume 25 µL): Master Mix 2x, forward and reverse primers in final concentration 0.5 µM and 37.5 ng DNA (final concentration 1.5 ng/µL).

The obtained results of digested and undigested DNA were obtained as x-fold of the relative quantification to *actin* (the reference gene). The pattern of gene methylation sites was calculated according to the equations: CCGG (non-methylated cytosines) = total DNA – DNA left after digest by *HpaII*; CCmGG (internal methylated cytosine) = DNA left after digest by *HpaII* - DNA left after digest by *MspI*; CmCmGG (external and internal methylated cytosine) = DNA left after digest by *MspI*. The changes in the different methylated cytosine in DNA (CCGG, CCmGG, and CmCmGG) were presented as x-fold relatively to the control.

Seven CCGG sites were analyzed (three in the promoter (P) and three in the exon (E) and one in the intron (I)) in the *β-1,3-glucanase 1* gene, twelve sites (eleven in the exon and one in the intron) in the *β-1,3-glucanase 2* gene, and eight sites in the exon in the *chitinase* gene. The *β-1,3-glucanase 1* gene sites were characterized by the following positions starting from ATG in DNA: P1 -1204-1201; P2 -1147-1144; P3 -344-341; E1 + 440-443; E2 + 1479-1482; E3 + 1763-1766; I1 + 2341-2344; the *β-1,3-glucanase 2* gene: E1 +50-53; E2 +432-435; E3 +690-693; E4 +883-886; E5 +891-894; E6 +1009-1012; E7 +1024-1027; E8 +1034-1037; E9 +1097-1100; E10 +1178-1181; E11 +1421-1424; I1 +1874-1877; and the *chitinase* gene: E1 +118-121; E2 +138-141; E3 +163 – 167; E4 +227-230; E5 +401-404; E6 +467-470; E7 +957-960; E8 +1105-1108. A schematic diagram of the position of all putative sites and the relative position of the primers that were used to perform qPCR to estimate methylation levels is presented in Figure S2.

### 2.5. Determination of Total DNA Methylation

The global level of DNA methylation of flax infected by *Fusarium* strains was determined colorimetrically by measuring the level of 5-methylcytosine using the dedicated MethylFlash Methylated DNA Quantification Kit (EpiGentek Group Inc., Farmingdale, NY, USA) according to the manufacturer’s instructions.

### 2.6. Gene Expression Analysis

The level of transcript for PR genes (*β-1,3-glucanase 1*, *β-1,3-glucanase 2*, *chitinase*), genes involved in chromatin modification (*chromomethylase 1*, *chromomethylase 3*, *repressor of silencing 1*, *demeter*, *argonaute 1*, *argonaute 4*, *RNA-dependent RNA polymerase 2*), and *actin* gene was measured using the quantitative PCR technique. 

The total RNA was isolated from plant tissues by treatment with Trizol reagent (Life Technologies, Carlsbad, California, USA) according to the manufacturer’s manual. Its quality was verified by gel electrophoresis on 1.5% (w/v) agarose containing 15% (v/v) formaldehyde and quantitated spectrophotometrically (Spectrophotometer Implen NanoPhotometer Pearl, München, Germany) at λ = 260 nm. In order to remove the residual genomic DNA, the samples were subjected to digestion with DNase I (Thermo Fisher Scientific, Waltham, Massachusetts, USA). Then, purified RNA served as a template for reverse transcription PCR to obtain cDNA. The reaction was designed using a High Capacity cDNA Reverse Transcription Kit (Applied Biosystems, Foster City, California, USA).

The quantitative PCR reactions were run using a SYBR dye-based set of reagents (DyNAmo HS SYBR Green qPCR Kit, Thermo Fisher Scientific, Waltham, Massachusetts, USA) on an Applied Biosystems StepOnePlus Real-Time PCR System (Life Technologies, Carlsbad, California, USA). Primers were designed to selectively amplify only plant sequences (not fungal, see Table S1) using LightCycler Probe Design Software 2 (Roche, Basel, Switzerland). Their specificity at annealing temperature was verified by analyzing the melting curves of the obtained reaction products. The reaction setup was designed according to the kit manufacturer’s protocol. The program used for real-time PCR was 95 °C for 10 min, and 40 cycles of denaturation for 15 s at 95 °C, annealing for 20 s at 57 °C, and extension for 30 s at 72 °C, and reaction mixtures contain (in final volume 25 µL): Master Mix 2x, forward and reverse primers in final concentration 0.5 µM and 125 ng cDNA (final concentration 5 ng/µL) All the reactions were run in triplicate. Changes in gene expression levels are presented as x-fold of relative quantities (RQ) standardized for *actin* (the reference gene) in relation to the control, non-treated plants.

### 2.7. Statistical Analysis

All experiments were repeated three times in separate runs. The data are shown as mean values ± standard deviation. The Statistica 7 (StatSoft, USA) software was used to estimate statistical significance of the data by means of Student’s t test. For expression change analysis one-sample t-test was performed on logFC values. The *p*-values are specified separately for each data set (* *p* < 0.05).

## 3. Results

### 3.1. DNA Methylation Patterns of β-1,3-Glucanase and Chitinase Genes

**Figure 1:**
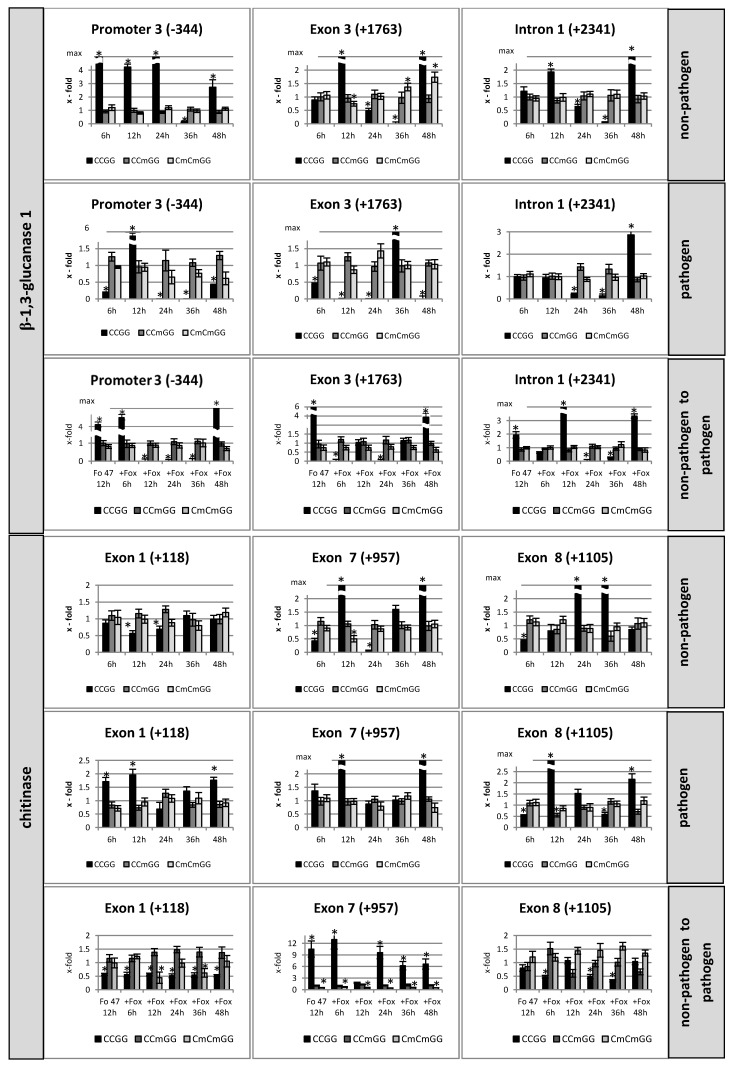
Changes in DNA methylation pattern of *β-glucanase 1* and *chitinase* in flax seedlings treated with non-pathogenic or pathogenic strains of *Fusarium oxysporum* and after sensitizing effects of the non-pathogenic strain treated with pathogenic Foln3 at 6, 12, 24, 36, and 48 h after inoculation presented as x-fold change in relation to control. The analysis of the third position in the exon, the first in the intron, and the third in the promoter of *β-1,3-glucanase 1* and the first, seventh, and eighth positions in the exon of *chitinase* was performed by the digestion of genomic DNA by the restriction enzymes *HpaII* and *MspI* and then the real-time PCR reaction. The data represent the mean from three biological repetitions. Asterisks mark statistically significant differences (*p* < 0.05) between the treated samples and their own controls.

Due to the lack of flax genes’ annotation in a database, in the first step we had to extract whole gene sequences for two isoforms of *β-1,3-glucanase* and *chitinase* genes on the basis of known gene fragments to find whole DNA sequences. The obtained sequences were analyzed in silico in order to identify the potential methylation sites. We found 7 CCGG sites in the *β-1,3-glucanase 1* gene (three in the promoter, three in exons, and one in the intron), 12 CCGG sites in the *β-1,3-glucanase 2* gene (eleven in exons and one in the intron), and 8 CCGG sites in exons in the *chitinase* gene. The analysis of methylation profiles of PR genes included all potential CCGG sites. Here we present only data for those sites where differences in a methylation pattern were observed between treatments. The differentiating CCGG sites for the β-1,3-glucanase 1 gene (3 sites) and chitinase gene (3 sites) are shown as x-fold of different methylation of CCGG (CCGG, CCmGG, and CmCmGG) in Figure 1 and as the DNA methylation pattern in Figure S3. We found that the methylation in all analyzed CCGG sequences for the *β-1,3-glucanase 2* gene did not change. 

The DNA methylation pattern of the *β-1,3-glucanase 1* gene in flax incubated with the non-pathogenic strain of *Fusarium oxysporum* was characterized by changes in the level of methylation of internal and/or external cytosine in three CCGG sequences (Figure 1). Analyzing the third CCGG place in the promoter of the *β-1,3-glucanase 1* gene, it was observed that the level of non-methylated cytosines (CCGG) significantly increased over 2.7-fold at the 6^th^, 12^th^, 24^th^, and 48^th^ hour of incubation and decreased to 12% at the 36^th^ hour. The levels of internal methylated cytosine (CCmGG) and internal and external methylated cytosines (CmCmGG) did not change significantly in flax plants incubated with the non-pathogenic strain of *F. oxysporum*. Other changes were shown for the third place in the exon, where a significant increase in non-methylated cytosines (CCGG) was observed at the 12^th^ and 48^th^ hour after incubation, a reduced level to 50% at the 24^th^ hour, and a lack of non-methylated cytosines at the 36^th^ hour. It was followed by a decrease at the 12^th^ hour in the CmCmGG and 1.4-fold and 1.7-fold increases at the 36^th^ and 48^th^ hour, respectively. It was interesting that for CCGG islands located in the intron, the level of CCmGG and CmCmGG remained unchanged, but the level of non-methylated cytosines increased over 1.9-fold at the 12^th^ and 48^th^ hour of incubation and decreased to 64% at the 24^th^ and 7% at the 36^th^ hour. 

The analysis of eight CCGG places in the *chitinase* gene revealed that only three of them were characterized by changes in the DNA methylation pattern in flax incubated with the non-pathogenic strain of *Fusarium oxysporum* (Figure 1). In the first CCGG site in the exon, the level of non-methylated cytosines decreased to 56% and 68% at the 12^th^ and 24^th^ hour of incubation with the non-pathogenic strain. An increase in non-methylated cytosines in the CCGG site was noted in the seventh exon (10-fold increase at the 12^th^ hour and more than 6-fold increase at the 48^th^ hour of incubation). It was followed by a decrease in the CCGG to 43% and 8% at the 6^th^ and 24^th^ hour, respectively. In addition, in the eighth site in the exon, the largest increase of the non-methylated cytosines in the CCGG was observed at the 24^th^ and 36^th^ hour after incubation together with the reduced level of the CCGG to 42% at the 6^th^ hour. 

In flax plants incubated with the pathogenic strain of *F. oxysporum* the profile status of methylation of cytosines in CCGG sites of the *β-1,3-glucanase 1* gene differed from that determined for flax incubated with the non-pathogenic strain, but the same differentiating CCGG sites remained. We observed a reduced level of non-methylated cytosines at the 6^th^, 24^th^, 36^th,^ and 48^th^ hour (a reduction to 20% and to 42% at the 6^th^ and 48^th^ hour and a total reduction at the 24^th^ and 36^th^ hour) and its significantly higher level at the 12^th^ hour in the third CCGG place in the promoter. In the third place in the exon a reduced level of non-methylated cytosines in CCGG was observed at the 6^th^, 12^th^, 24^th,^ and 48^th^ hour of incubation (a reduction to 43% at the 6th and a total reduction at the 12^th^, 24^th^, and 48^th^ hour) and a significant increase at the 36^th^ hour. In the intron, the decrease in non-methylated cytosines in the CCGG site to 21% and 14% at the 24^th^ and 36^th^ hour after incubation, respectively, was subsequently replaced by an increase (2.9-fold) in non-methylated cytosines at the 48^th^ hour. 

The first, seventh, and eighth CCGG sites in the exons in the *chitinase* gene are differentiating sites in flax inoculated with pathogen. A thorough analysis of these CCGG sites in the *chitinase* gene revealed about a two-fold increase in non-methylated cytosines at the 6^th^, 12^th^, and 48^th^ hour of incubation in the first CCGG site as well as the largest increase in non-methylated cytosines at the 12^th^ and 48^th^ hour in the seventh site. In the eighth CCGG site, we noted the largest increase in the non-methylated cytosines at the 12^th^ hour of incubation and also a significant but smaller increase at the 48^th^ hour of incubation. Additionally, the lower level of the non-methylated cytosines (a reduction to about 50%) at the 6^th^ and the 36^th^ hour after incubation was observed. The increase in the non-methylated cytosines at the 12^th^ hour was correlated with the decrease in the internal methylated cytosine at this time.

The methylation profile of the *β-1,3-glucanase 1* gene in flax seedlings incubated with the pathogenic strain after previous sensitization by Fo47 was determined based on an analysis of the third place in the promoter, the first place in the intron, and the third place in the exon (Figure 1). The third CCGG site in the promoter was characterized by a total reduced level of non-methylated cytosines at the 12^th^, 24^th^, and 36^th^ hour and a significant increase in non-methylated cytosines at the 6^th^ and 48^th^ hour. 

Moreover, the methylation profile of the *chitinase* gene was determined by analyzing the methylation status of eight CCGG sites in exons, of which three sites were altered (the first, seventh, and eighth). The level of non-methylated cytosines remained unchanged in relation to the sensitized plants in all time points investigated. However, we observed a reduced level (about 50%) of internal and external methylated cytosines (CmCmGG) at the 12^th^ and 36^th^ hour. The seventh CCGG site in the exon showed a considerable increase in the non-methylated cytosines at all the time-points but the 12^th^, where the increase was smaller. Constant, reduced levels (about 50%) of internal and external methylated cytosines (CmCmGG) were observed. The eighth site in the exon was characterized by a reduced level of non-methylated cytosines at the 6^th^, 24^th^, and 36^th^ hour.

### 3.2. Level of Total DNA Methylation

**Figure 2:**
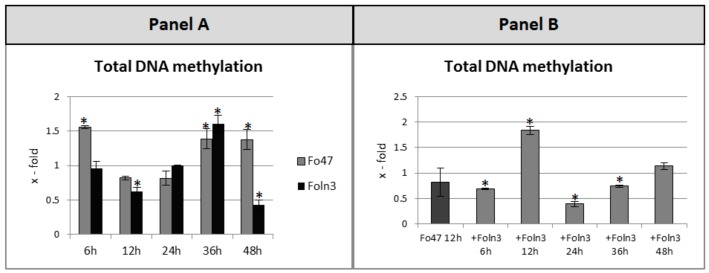
Total DNA methylation in flax seedlings treated with non-pathogenic (Fo47) or pathogenic strains of *Fusarium oxysporum* (Foln3) (panel **A**) and after sensitizing effects of the non-pathogenic strain treated with pathogenic Foln3 (+ Foln3) (panel **B**) at 6, 12, 24, 36, and 48 h after inoculation. The data represent the mean from three biological repetitions. Asterisks mark statistically significant differences (*p* < 0.05) between the treated samples and their own controls.

After determining the changes in DNA methylation pattern of *β-glucanase 1* and *chitinase* in flax seedlings incubated with non-pathogenic and pathogenic strains of *F. oxysporum* and in flax infected by *Foln3* after previous treatment with the non-pathogenic strain we examined the total level of DNA methylation, and the results are shown in Figure 2.

The level of total DNA methylation increased by about 1.5-fold at the 6^th^, 36^th^, and 48^th^ hour of flax seedlings’ incubation with the non-pathogen, while at the other analyzed time points only a slight decrease (approximately 20%) was observed when compared to control plants. In the case of the flax plants incubated with the pathogenic strain, a 1.6-fold increase in total DNA methylation at the 36^th^ hour and a reduction to 62% and 43% at the 12^th^ and 48^th^ hour of incubation with the pathogen, respectively, were noted. However, in the flax sensitized with Fo47 the total level of DNA methylation increased 1.8-fold at the 12^th^ hour of incubation, and was reduced to 70%, 40%, and 74% at the 6^th^, 24^th^, and 36^th^ hour, respectively.

### 3.3. Expression Levels of PR Genes

**Figure 3:**
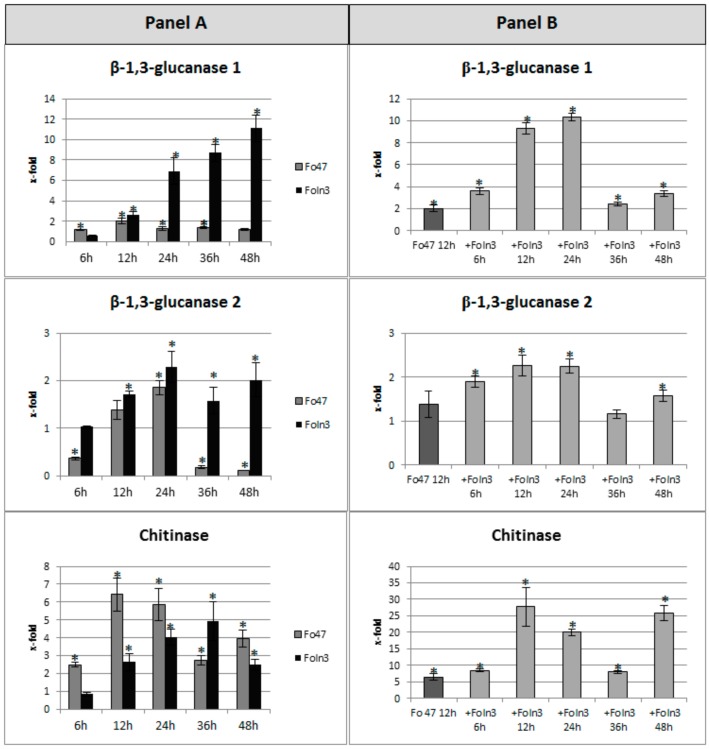
Expression level of *β-glucanase 1, β-glucanase 2,* and *chitinase* in flax seedlings treated with non-pathogenic (Fo47) or pathogenic strains of *Fusarium oxysporum* (Foln3) (panel **A**) and after sensitizing effects of the non-pathogenic strain treated with pathogenic Foln3 (+ Foln3) (panel **B**) at 6, 12, 24, 36, and 48 h after inoculation. The data were obtained from real-time RT-PCR analysis. *Actin* was used as a reference gene and the transcript levels were normalized to the control plant. The data represent the mean ± standard deviations from three biological repetitions. Asterisks mark statistically significant differences (*p* < 0.05) between the treated samples and their own controls.

The expression levels of PR genes, two isoforms of *β-1,3-glucanase* and one of *chitinase,* were determined in flax seedlings treated with non-pathogenic and pathogenic strains of *Fusarium oxysporum* as well as in flax seedlings sensitized by the non-pathogenic strain Fo47 and then incubated with the pathogenic strain Foln3. In order to present the precise kinetics of changes, the analyses were performed after 6, 12, 24, 36, and 48 h of flax incubation with appropriate *Fusarium* strains and the obtained results are presented in Figure 3.

In flax seedlings incubated with the non-pathogenic strain of *Fusarium oxysporum,* the analysis of the expression level of *β-1,3-glucanase 1* gene revealed a two-fold increase at the 12^th^ hour of incubation. An increase in the transcript level was also noted at subsequent time points (the 24^th^ and 36^th^ hour), but it was lower compared to the earlier time (the 12^th^ hour). In the case of flax incubated with the pathogenic strain, the expression level of the *β-1,3-glucanase 1* gene increased continuously with the incubation time (from a 2.6-fold increase at the 12^th^ hour to 11-fold at the 48^th^ hour). However, in the sensitized flax, the transcript level of the *β-1,3-glucanase 1* gene increased during incubation from 3.6-fold at the 6^th^ hour to 10.3-fold at the 24^th^ hour and then decreased to the initial level (after 12 h priming) and then finally a 3.4-fold increase at the 48^th^ hour was observed.

In flax plants incubated with the non-pathogenic strain, the second isoform of the *β-1,3-glucanase* gene was characterized by a 1.85-fold increase in expression level after 24 h of incubation and a decrease to 65%, 20%, and 12% at the 6^th^, 36^th^, and 48^th^ hour, respectively. Furthermore, the analysis of this gene in flax seedlings incubated with the pathogenic strain revealed the smallest changes in the expression level as compared to other PR genes. However, in comparison with control plants the mRNA level of this gene increased 1.7-fold, 2.3-fold, 1.6-fold, and 2-fold at the 12^th^, 24^th^, 36^th^, and 48^th^ hour of incubation with pathogenic *F. oxysporum*, respectively. In contrast, the incubation of flax seedlings with the pathogen after previous sensitization by the non-pathogenic strain had a slight effect on the mRNA level of the *β-1,3-glucanase 2* gene. A significant increase in the expression of this gene (about 2-fold) occurred at the 6^th^, 12^th^, and 24^th^ hour of incubation with the pathogenic strain and 1.6-fold increase at 48^th^ hour was observed.

For the *chitinase* gene, an increase in the mRNA levels at each time point after incubation with the non-pathogenic strain was observed, with the largest increase at the 12^th^ and 24^th^ hour of incubation (6.4-fold and 5.8-fold increase, respectively). In flax seedlings incubated with the pathogenic strain, the transcript level of *chitinase* increased from 2.6-fold at the 12^th^ hour to 4.9-fold at the 36^th^ hour and then decreased, but still remained higher compared to the control (2.5-fold increase). However, flax treatment with pathogenic Foln3, after earlier sensitization with non-pathogenic Fo47, initially caused a significant 8.5-fold increase in *chitinase* gene expression at the 6^th^ hour, 27.7-fold increase at the 12^th^ hour, and a 20-fold increase at the 24^th^ hour. Then, it decreased to 8-fold of the control at the 36^th^ hour, and finally a large, 25.8-fold increase in the expression of *chitinase* at the 48^th^ hour was observed.

### 3.4. Expression Levels of Genes Involved in DNA Methylation

**Figure 4:**
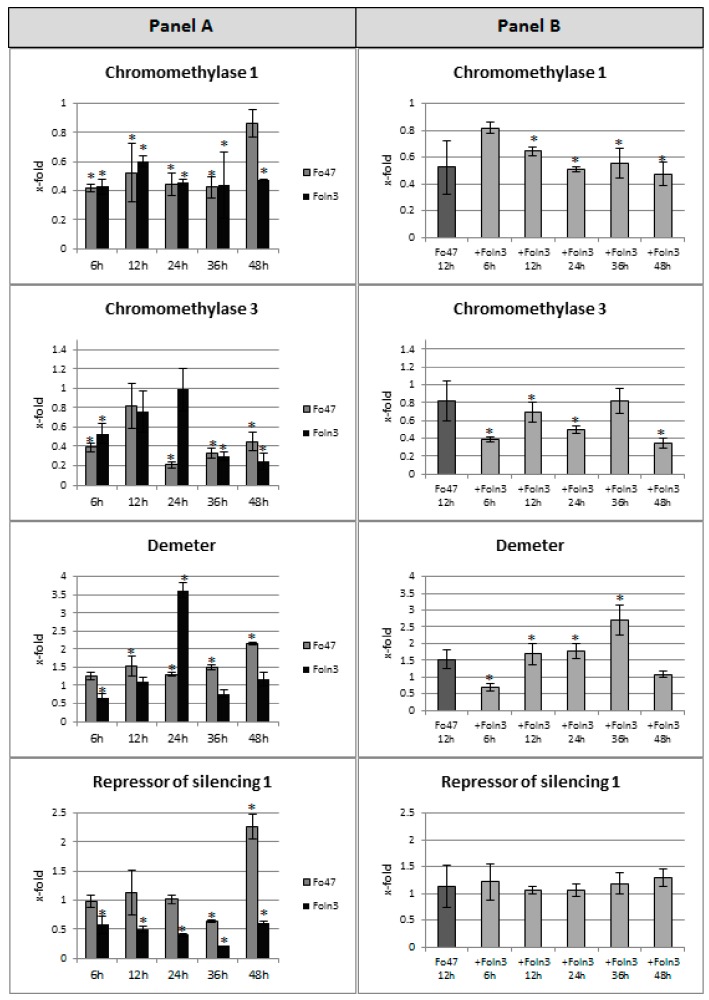

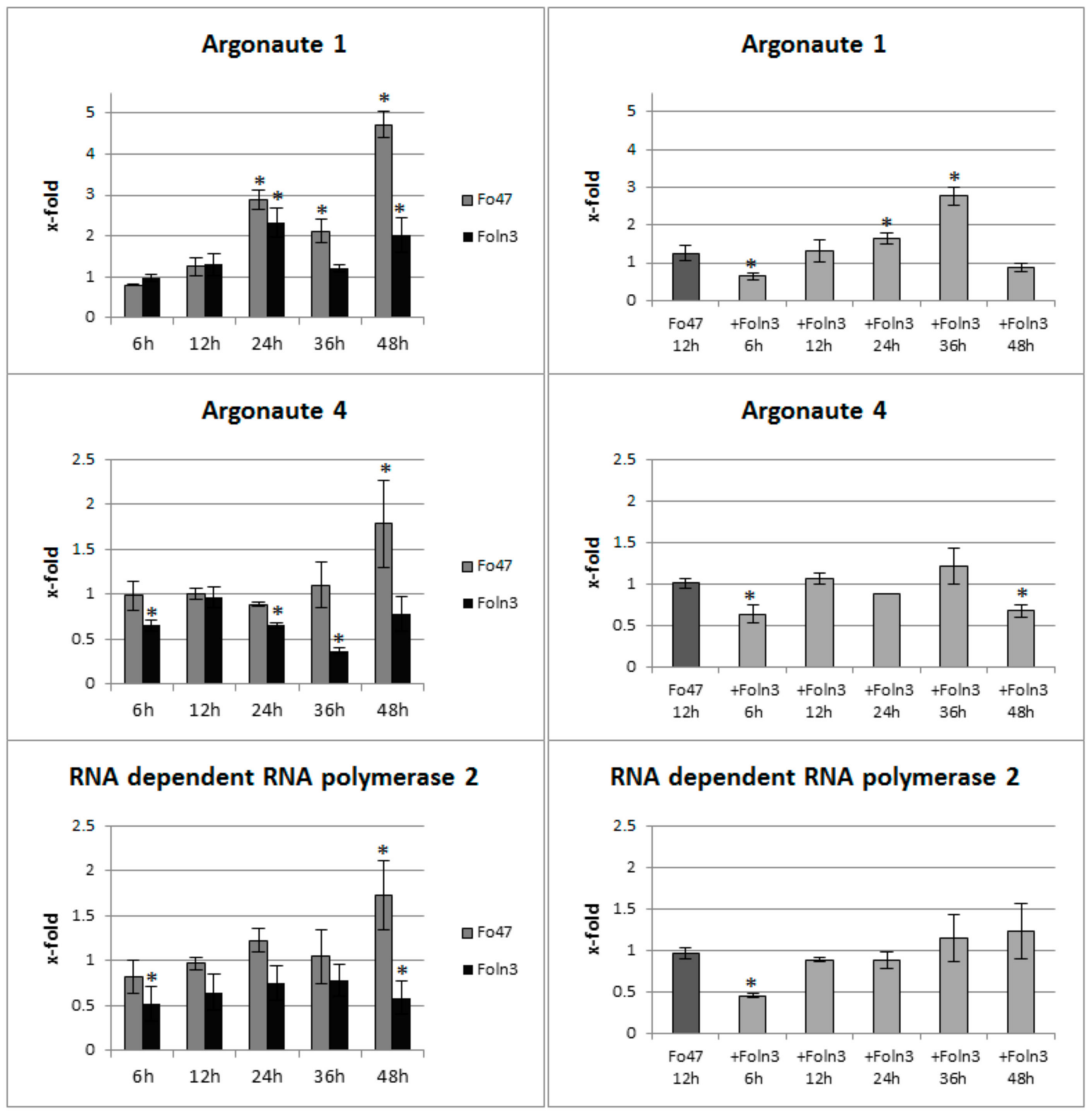
Expression levels of genes involved in DNA methylation (*chromomethylase 1*, *chromomethylase 3*, *demeter*, *repressor of silencing 1*, *argonaute 1*, *argonaute 4*, *RNA-dependent RNA polymerase 2*) in flax seedlings treated with non-pathogenic (Fo47) or pathogenic strains of *Fusarium oxysporum* (Foln3) (panel **A**) and after sensitizing effects of the non-pathogenic strain treated with pathogenic Foln3 (+ Foln3) (panel **B**) at 6, 12, 24, 36, and 48 h after inoculation. The data were obtained from real-time RT-PCR analysis. *Actin* was used as a reference gene and the transcript levels were normalized to the control plant. The data represent the mean ± standard deviations from three biological repetitions. Asterisks mark statistically significant differences (*p* < 0.05) between the treated samples and their own controls.

Due to the changes in methylation profiles of PR genes and the need to explain such changes, the next stage of the study was to analyze the expression of genes involved in the DNA modifications: Methylation (*chromomethylase 1, CMT1* and *chromomethylase 3*, *CMT3*) and demethylation (*repressor of silencing 1*, *ROS1* and *demeter*, *DME*). Additionally, *argonaute 1* (*AGO1*), *argonaute 4* (*AGO4*), and *RNA dependent RNA polymerase 2* (*RDR2*) genes involved in mechanisms of DNA modification were assessed. Changes in expression levels of genes analyzed in flax seedlings incubated with non-pathogenic and pathogenic strains of *F. oxysporum* and in flax incubated with Foln3 after having a sensitizing effect by Fo47 are shown in Figure 4.

In flax incubated with the non-pathogenic *F. oxysporum*, expression levels of *CMT1* and *CMT3* genes were decreased: A reduction to 40% at the 6^th^ hour of incubation. Additionally, a lower expression level of the *CMT1* gene was maintained until the 36^th^ hour. In contrast, the expression level of the *CMT3* gene was increased at the 12^th^ hour of incubation (up to a level equal to the control), but it again decreased by 79%, 67%, and 55% at the 24^th^, 36^th^, and 48^th^ hour, respectively. The *DME* gene exhibited a slight (1.25- to 1.5-fold) increase in the transcript level over time of the flax incubation with the non-pathogenic strain of *Fusarium*, and finally, at the 48^th^ hour, the expression level of this gene increased more than two-fold. An analysis of a second gene involved in DNA demethylation (*ROS1*) revealed a decrease in the expression level of this gene to 63% at the 36^th^ hour and a 2.3-fold increase at the 48^th^ hour compared to the control. Furthermore, the incubation of flax with the non-pathogenic strain of *Fusarium* affected the expression of genes involved in other mechanisms that modify the DNA. Expression of *AGO4* and *RDR2* genes increased (1.8-fold and 1.7-fold, respectively) at the 48^th^ hour of incubation, while at earlier time points it did not change at all. The *AGO1* gene was characterized by greater variability, wherein the level of expression was increased 2.9-fold, 2.1-fold, and 4.7-fold at the 24^th^, 36^th^, and 48^th^ hour, respectively.

After incubation of flax plants with the pathogenic *F. oxysporum*, we observed a decrease in the expression of genes involved in the process of DNA methylation. The transcript level of the *CMT1* gene was reduced to approximately 50% for all analyzed times, while the transcript level of the *CMT3* gene was reduced to 50% at the 6^th^ hour, then it returned to the control level at the 12^th^ hour and 24^th^ hour, and then decreased to 30% at the 36^th^ and 48^th^ hour. The DNA demethylation genes responded differently in flax incubated with the pathogen. The expression level of the *ROS1* gene was about half of the control at the majority of time points, with the lowest value (a reduction to 20%) at the 36^th^ hour. The level of expression of the *DME* gene significantly increased by 3.6-fold at the 24^th^ hour, but was also slightly reduced to 65% at the 6^th^ hour. As in the case of the treatment of flax seedlings with the non-pathogen, flax incubation with the pathogen induced similar changes in the expression of *AGO4* and *RDR2* genes and various changes of the *AGO1* gene. The transcript levels of *AGO4* genes were reduced to 50–75% during incubation with pathogen. Only 12 and 48 h after incubation did the mRNA level of the *AGO4* gene remain unchanged. The level of mRNA of the *RDR2* gene was reduced to 51% and 58% at the 6^th^ and 48^th^ hour, respectively. Similarly to the non-pathogenic strain of *Fusarium*, the pathogenic strain caused a 2.3-fold increase in expression of the *AGO1* gene at the 12^th^ hour and a two-fold increase after 48 h of incubation.

In flax incubated with pathogenic Foln3 after prior sensitization by non-pathogenic Fo47 the expression levels of *CMT1* and *CMT3* genes differed significantly from each other, which was not observed in flax after incubation either with pathogenic or with non-pathogenic *F. oxysporum*. The expression of the *CMT1* gene was reduced below 80% in all analyzed time points. The second gene, *CMT3*, showed a decrease in the level of mRNA (to about 40% at the 6^th^, to 70% at the 12^th^, to 50% at the 24^th^, and to 30% at the 48^th^ hour). Among genes participating in DNA demethylation, only the transcript level of the *DME* gene changed, whereas the mRNA level of *ROS1* remained unchanged. The expression level of *DME* declined to 70% at the 6^th^ hour; then showed a 1.7-fold, 1.8-fold and 2.7-fold increase at the 12^th^, 24^th^ and 36^th^ hour. The mRNA level of the *RDR2* gene was reduced to 40% at the 6^th^ hour, but starting from the 12^th^ hour a return to the state before incubation was observed. A decrease in the expression was also noted for the *AGO4* gene (to 60% and 70% at the 6^th^ and 48^th^ hour, respectively). The *AGO1* gene showed another pattern of expression, wherein initially at the 6^th^ hour of incubation there was a 40% decrease in expression level, then a 1.6-fold and a 2.8-fold increase at the 24^th^ and 36^th^ hour, and a drop back to 90% compared to the control.

### 3.5. Phenotypic Changes in Flax after Sensitization through Non-Pathogenic Strain of F. Oxysporum

The additional step in evaluation of the sensitizing action of the *Fusarium oxysporum* non-pathogenic strain on flax was based on the phenotypic analysis of plants from in vitro cultures, which were first incubated for two days with the non-pathogenic strain of *Fusarium oxysporum* (for comparison, plants were also incubated without fungi (control plants)) and then through the subsequent days with the pathogenic strain (as previously, appropriate controls were prepared). The first pictures of plants were taken after four and six days (Figure S4). 

It is visible at 4 days after inoculation that the prior plant incubation with the non-pathogenic strain reinforced the resistance to pathogen infection compared to plants which had not been pre-incubated with the non-pathogenic strain. These differences increased at later time points. It seems that the selected two-day period of sensitization was optimal for the plants. Plants that have been incubated for the first two days and a further four and six days on medium with the pathogen looked the worst.

The phenotypic analysis of flax after the sensitizing action caused by the non-pathogenic strain of *F. oxysporum* and then after incubation with pathogenic *F. oxysporum* confirmed our supposition that non-pathogenic strains could enhance the flax plants’ resistance to the pathogen infection.

## 4. Discussion

The study of changes in the level of genome methylation mostly accompanied plant development and plant response to biotic and abiotic stressors. In the case of environmental stresses, they can affect methylation in two ways, causing hyper- or hypomethylation. For example, differences in plant methylomes have been found during maize leaf development [24] and precede transcriptional activation of genes leading to cell division and meristem growth in potatoes [25]. Also, endosperm and embryo development [26], vernalization, and fruit ripening are affected by DNA methylation [27]. For example, rice blight pathogen resistance is related to genomic hypermethylation and hypomethylation of resistance-related genes [28]. Also, Infection of tobacco with tobacco mosaic virus (TMV)revealed an increase in genomic methylation and hypomethylation of resistance-related leucine-rich-repeat (LRR)-containing loci [29]. The data thus suggest that DNA methylation affects the plant genome not only at the global level but also at very specific sites, such as individual genes [30]. The identification of differentially methylated CCGG sites within pathogenesis-related (PR) genes: *β-1,3-glucanase* and *chitinase*, the time course of their modification, and the correlation with their expression profile in plants remain largely unexplored. Here we analyzed the methylation state of all CCGG sites of three flax genes: Two *β-1,3-glucanase* (*1* and *2*) and *chitinase* genes. It was found that three CCGG sites of the *β-1,3-glucanase 1* gene (located in promoter, exon, and intron regions) and *chitinase* were changed upon infection and these were further analyzed. The tetramer methylation and expression of *β-1,3-glucanase 2* change slightly upon infection, and served for comparison. The *β-1,3-glucanase 1* and *chitinase* appeared to be strongly activated upon pathogen infection. The analysis revealed that the level of unmethylated sites CCGG ranged from 0 to 19%, CCmGG methylation occurred in 75% on average, and the CmCmGG methylation level was around 15% of all investigated CCGG sites. In contrast to the developing maize leaf where *HpaII*/*MspI* restriction analysis of the CCGG site revealed that almost half (48.1%) of the loci were unmethylated, full CG methylation was represented by 36.7% and CHG hemimethylation by 15.2% of all investigated CCGG sites [31].

As mentioned, two types of CCGG sites in *β-1,3-glucanases* have been detected: Those whose methylation status is unchanged and those with a highly modified cytosine state. The profile of the latter was changing upon plant growth and progression of the infection. It is in agreement with several observations that plants as sessile organisms have developed prompt response mechanisms to react to rapid environmental changes. It is interesting that the CCGG site methylation level induced by both pathogenic and non-pathogenic *Fusarium* strains is time dependent. After artificial inoculation with both *Fusarium* strains, the methylation pattern of the pathogen-responsive *β-1,3-glucanase* and *chitinase* genes undergoes rapid (6–24 h after inoculation) and dynamic changes in tetramer methylation. It was found that both strains induced a similar time-dependent profile of tetramer methylation but they differ significantly at the beginning and at the end of the profiles. Far earlier pulse demethylation of the *β-1,3-glucanase 1* gene (promoter) occurs and it is maintained longer upon non-pathogenic strain treatment than in the case of the pathogenic one. This suggests that the same CCGG site within the gene might be differentially methylated. Similar to this was the observation that in two cases, two different CCGG sites within the same gene were found to be differentially methylated. In the first one, a conserved C-terminal domain CTC-interacting domain-encoding gene (human ATAXIN2 orthologue) was hypermethylated in two exons, and in the second case a gene encoding the protein kinase PK/UbiC was differentially methylated at two consecutive CCGG sites in an exon and an intron [31].

The *chitinase* gene’s tetramers located at the beginning and end of the coding sequences (exons 1, 7, 8) are also pulse demethylated. Notably, in both genes the exon peak demethylation is reached earlier upon pathogen infection. These findings agree with observations that in maize the majority of the differentially methylated CCGG sites that map to a gene lie within an exon and they are not distributed equally throughout the gene body. The highest number of differentially methylated sites was in the first 10% and the last 20% of the gene body. In addition, differential methylation of the promoter and 5’ part of the gene anticorrelated with the gene expression while differential methylation of the central and 3’ part of the gene body or sequences downstream of the gene was unrelated to the gene expression. [31].

In *Arabidopsis thaliana*, promoter-specific methylation occurs in less than 5% of genes, most of which are under tissue-specific control. A surprising result of genome-wide methylation profiling revealed that about one-third of all genes contain CG-specific genic or body methylation patterns within their transcribed regions which are highly expressed [32]. However, it was indicated that in *A. thaliana,* genes with the highest and the lowest transcription level were the least methylated, while moderately transcribed genes were the most frequently methylated [33]. The correlation between expression levels and DNA methylation levels in the gene body region was also observed in *Populus trichocarpa* where the hypermethylation of genes caused the reduced level of their expression while the hypomethylation of genes led to the increased level of their expression [34]. Due to the lack of unambiguous literature data, there are still many questions about the gene body methylation function and its correlation with gene expression.

The levels of *β-1,3-glucanase 1* and *chitinase* gene expression are also significantly altered, while in the case of *β-1,3-glucanase 2* only slight changes were noted. This is consistent with the hypothesis that pathogen infection changes the methylation level of plant genomic DNA and leads to alterations in gene transcription. The peak of demethylation might be a signal for the alteration in gene expression induced by the non-pathogenic strain. In the case of pathogen infection the expression peak lags behind the gene demethylation: Maximal transcript accumulation was detected 24–36 h after the onset of peak methylation.

The result might suggest that flax treatment with the non-pathogenic strain prepares plants for reaction after contact with pathogen. Thus, following non-pathogenic pretreatment, plants were infected with the pathogenic strain, and gene expression was analyzed. Indeed, upon infection, the highest gene expression was reached far earlier in the case of plants pretreated with the non-pathogenic strain. The interesting fact is that the level of gene induction by the pathogen is 2-fold lower in the case of pretreated plants, but even so, they are efficiently protected against infection. While plants infected with the pathogen started to die (at 48 h), the pretreated plants grew normally.

We measured the expression of genes involved in this process and found that both *AGO4* and *RDR2* are activated upon non-pathogenic *Fusarium* treatment while they are suppressed by the pathogenic strain. The gene induction and thus potent target methylation appeared later (at 48 h) than *β-1,3-glucanase* and *chitinase* gene demethylation and their expression increased. We deduced that non-pathogenic strain infection induced at first demethylation and gene up-regulation and at the end, after reaching a certain level of transcript accumulation, activated the re-methylation pathway and thus gene silencing. Temporary demethylation/remethylation serves as memorized experience and helps the plant to overcome pathogenic infection by a mechanism that is similar to immunization.

It was detected that the plant-specific methyltransferase expression profile showed significant suppression upon infection with both *Fusarium* strains. However, the pattern of DNA methylation is the result of co-operative or competing interactions of the methyltransferases and the silencing pathways which involves repressor of silencing (ROS1) and demeter (DME), which encode two closely related DNA glycosylase domain proteins [21]. In *Arabidopsis thaliana*, ROS1 and DME are required for release of transcriptional silencing of a hypermethylated transgene and activate the maternal expression of two genes silenced by methylation [35]. The expression profile of these genes in infected flax differs depending on *Fusarium* strain used and growth time. However, the ratio between the level of methyltransferase and repressor of silencing pathway gene expression promote CCGG sites’ demethylation.

**Figure 5:**
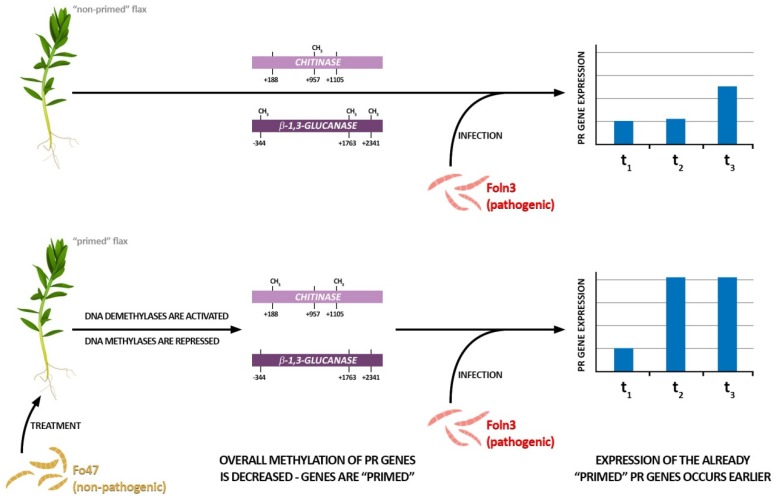
Hypothetical model of priming induced by non-pathogenic *Fusarium* strain. Flax treatment with non-pathogenic *Fusarium* strain (Fo47) results in changes in DNA methylation pattern of *β-1,3-glucanase* and *chitinase* genes which leads to their earlier expression during infection by pathogenic *Fusarium* strain (Foln3).

In summary, two contrasting effects on the levels of methylation in flax were detected upon infection of both *Fusarium* strains: Genome-wide hypermethylation and hypomethylation of two resistance-related genes, which resulted in an increase of their expression. Plants pretreated with the non-pathogenic strain memorized the hypomethylation pattern and then reacted more efficiently upon pathogenesis (Figure 5). However, the changes in methylation profile and thus the regulation of PR-related gene expression were not the only mechanisms of plant response to stressors. For example, it is known that the argonaute family proteins are core constituents of RNA interference pathways in eukaryotes and mediate gene expression. Guided by sRNAs, AGO proteins bind target sequences through base pairing and following recruitment of cofactors mediate post-transcriptional or transcriptional gene silencing. AGO proteins are also involved in epigenetic modifications of chromatin. In *Arabidopsis thaliana*, AGO4 guided by 24-nt small interfering RNAs (siRNA) recruits DNA methyltransferase for de novo DNA methylation at target loci [36]. Likewise, AGO1 with siRNAs facilitates H3K9 methylation by recruiting H3K9 methyltransferase [37].

Very recently, it was reported that direct binding of AGO1, guided by a 21-nt siRNA, to the chromatin of active genes promotes their transcription. Various stimuli, including plant hormones and stresses, specifically trigger AGO1 guided by siRNAs to bind stimulus-responsive genes [38]. Here, we found strong up-regulation of *AGO1* expression in *Fusarium*-treated plants. It thus suggests that in addition to the transcriptional regulation of gene expression, microRNAs and siRNAs, epigenetics occurs as an important mechanism involved in transcriptional regulation of the plant stress response. These phenomena may contribute to the adaptation of plants to the environment and stress situations.

## Supplementary Materials

The following are available online at https://www.mdpi.com/2076-2607/7/12/589/s1, Figure S1: A simplified scheme of the treatment of flax plants with the pathogenic and non-pathogenic strain of *F. oxysporum* and the treatment of flax plants with pathogenic strain after sensitizing with a non-pathogenic strain; Figure S2: Schematic diagram of the position of all CCGG sites and the relative position of the primers in *β-1,3-glucanase* 1, *β-1,3-glucanase 2* and *chitinase* genes that were used to perform qPCR to estimate methylation levels; Figure S3: Changes in DNA methylation pattern of *β-glucanase 1* and *chitinase* genes in flax seedlings treated with non-pathogenic or pathogenic strains of *Fusarium oxysporum* and after sensitizing effects of the non-pathogenic strain treated with pathogenic Foln3 at 6, 12, 24, 36 and 48 h after inoculation. The analysis of the third position in the exon, the first in the intron and the third in the promoter of *β-1,3-glucanase 1*, and the first, seventh and eight positions in the exon of *chitinase* was performed by the digestion of genomic DNA by the restriction enzymes *HpaII* and *MspI* and then the real-time PCR reaction. C, control; N-P, non-pathogen; P, pathogen; CCGG, sequence where cytosines are not methylated; CCmGG, sequence where internal cytosine is methylated; CmCmGG, sequence where two cytosines are methylated. CCGG positions in DNA are counted from ATG. The data represent the mean from three independent experiments. Asterisks mark statistically significant differences (*p* < 0.05) between the treated samples and their own controls; Figure S4: Phenotypic changes in flax after priming of the non-pathogenic strain of *Fusarium oxysporum* treated with pathogenic Foln3. Flax plants from in vitro culture were grown for 2 days on PDA medium or on PDA medium with a non-pathogenic (Fo47) or pathogenic (Foln3), and then were transferred to control PDA medium or PDA medium with Foln3 or Fo47 for 6 days. Analyzed combinations: control to control, control to Foln3, Fo47 to Foln3, Fo47 to Fo47, Foln3 to Foln3; Table S1: Primer sequences designed for real-time PCR: (A) PR genes (*β-1,3-GLU1*, *β-1,3-glucanase 1*; *β-1,3-GLU2*, *β-1,3-glucanase 2*; *CHIT*, *chitinase*), genes involved in chromatin modification (*CMT1*, *chromomethylase 1*; *CMT3*, *chromomethylase 3*; *ROS1*, *repressor of silencing 1*; *DME*, *demeter*; *AGO1*, *argonaute 1*; *AGO4*, *argonaute 4*; *RdRp2*, *RNA-dependent RNA polymerase 2*) and *actin* gene. (B) DNA methylation. E3-G1 (the third position in the exon of *β-1,3-glucanase 1*); I1-G1 (the first position in the intron of *β-1,3-glucanase 1*); P1-G1 (the third position in the promoter of *β-1,3-glucanase 1*); E1-G2 (the first position in the exon of *β-1,3-glucanase 2*); E11-G2 (the eleventh position in the exon of *β-1,3-glucanase 2*); I1-G2 (the first position in the intron of *β-1,3-glucanase 2*); E1-CH (the first position in the exon of *chitinase*); E7-CH (the second position in the exon of *chitinase*); E8-CH (the eighth position in the exon of *chitinase*).
